# Psychological trauma and digital interventions: myth of reality?

**DOI:** 10.1192/j.eurpsy.2023.149

**Published:** 2023-07-19

**Authors:** I. Frankova

**Affiliations:** Clinical, Neuro- and Developmental Psychology, Vrije Universiteit Amsterdam, Amsterdam, Netherlands

## Abstract

**Abstract:**

The war in Ukraine caused many people to flee within Ukraine and across Europe. Trauma, separation from family and prolonged uncertainty increase the risk of posttraumatic stress disorder and depression among Ukrainians. The element of time is a critical aspect of intervention in stress-related disorders as an identical intervention may be beneficial or harmful if given at different times, focussing on primary, secondary or tertiary prevention. Recent studies showing the importance of timing of intervention in prevention of PTSD lend further support to the concept of the “golden hours” or “window of opportunity”. Dr. Iryna Frankova will present lessons learned from implementation of this psychological first aid digital intervention of low intensity during the ’golden hours’ following escalation of the war in Ukraine. During the talk she will provide guidance on what should and should not be done for the end users, as well as strengths and limitations of the chatbot intervention.

**Disclosure of Interest:**

None Declared

